# The effects of background white noise on memory performance in inattentive school children

**DOI:** 10.1186/1744-9081-6-55

**Published:** 2010-09-29

**Authors:** Göran BW Söderlund, Sverker Sikström, Jan M Loftesnes, Edmund J Sonuga-Barke

**Affiliations:** 1 Department of Linguistics, Stockholm University, Sweden; 2 Linné University, Växjö, Sweden; 3 Sogn and Fjordane University College, Norway; 4 University of Southampton, Dept. of Psychology, UK; 5 Department of Experimental Clinical and Health Psychology, Ghent University, Belgium

## Abstract

**Background:**

Noise is typically conceived of as being detrimental for cognitive performance; however, a recent computational model based on the concepts of stochastic resonance and dopamine related internal noise postulates that a moderate amount of auditive noise benefit individuals in hypodopaminergic states. On the basis of this model we predicted that inattentive children would be enhanced by adding background white noise while attentive children's performance would deteriorate.

**Methods:**

Fifty-one secondary school pupils carried out an episodic verbal free recall test in two noise conditions. In the high noise condition, verb-noun sentences were presented during auditory background noise (white noise, 78 dB), and in the low noise condition sentences were presented without noise.

**Results:**

Exposure to background noise improved performance for inattentive children and worsened performance for attentive children and eliminated episodic memory differences between attentive and inattentive school children.

**Conclusions:**

Consistent with the model, our data show that cognitive performance can be moderated by external background white noise stimulation in a non-clinical group of inattentive participants. This finding needs replicating in a larger sample using more noise levels but if replicated has great practical applications by offering a non-invasive way to improve school results in children with attentional problems.

## Background

It has long been known that cognitive processing is easily disturbed by incompatible environmental stimulation which distracts attention from tasks [1]. This effect is believed to stem from competition for attentional resources between the distracting and the target stimuli. Such negative distractor effects hold across a wide variety of tasks and stimuli as well as in different participant populations [2-6]. For some populations the effects are predicted to be especially strong. For instance, individuals with attentional problems such as attention deficit/hyperactivity disorder (ADHD) are generally acknowledged to be more vulnerable to distraction than normal control children [7,8].

At the same time there are reports of contradictory findings where certain types of task irrelevant noise actually improve the performance of children. Surprisingly, this effect may also be most pronounced in children with attention deficits. Under certain circumstances children with attentional problems (including those with ADHD) benefit from, rather than being distracted by, background task-irrelevant noise presented concurrently with a target task. For instance, Stansfeld et al. [9] found that under certain conditions road traffic noise can improve performance on episodic memory tasks in children at risk for attentional problems and academic under-achievement. Research data from our group demonstrated that adding background white noise to the environment enhanced memory performance of children with ADHD [10]; although in every day situations optimal levels of white noise will vary from one individual to another.

Why these paradoxical effects should occur is not well understood. Most accounts in the past, for example the optimal stimulation theory by Zentall and Zentall [11] and later models of cognitive energetic and motivational processes [12], have focused on the role of background stimulation as a generator of increased arousal which counteracts boredom. A recent computational model has attempted to explain these positive effects of background noise on performance in a different way [13]. This model combines two factors: It explains (i) how noise enhances attention and performance in general by the concept of stochastic resonance (SR) and (ii) why there are individual differences in the way noise affects the brain by a model of individual differences in dopamine.

### Stochastic Resonance - how noise strengthens the signal

SR or noise-improved signaling is a well-established phenomenon across a range of experimental settings; SR exists in any threshold-based system with noise that requires a threshold to be passed before a signal is registered. SR can be observed in nature in any non-linear dynamic system, which is not working at its optimum level, in particular SR has been found in the nervous system. The simplest examples of an SR-related benefit can be seen in the detection of sensory signals. When a weak signal (e.g. a tone stimulus) is presented below the hearing threshold it becomes detectable when random or white noise is added to the signal. In essence, this account proposes that the additional variability provided by the noise interacts with the weak signal pushing it above the detection threshold, see review in [14]. For instance, SR has been found in several modalities; audition [15], vision [16], and touch [17] where stochastic noise improves sensory discriminability. Recently SR has been shown to work across modalities, for example when auditory noise improves visual signal detection [18]. Most SR studies have used perception tasks, requiring the detection of weak peripheral sensory inputs. Few studies have examined how noise influences cognitive performance. Recent empirical evidence suggests that SR can also improve central processing and cognitive performance. For example, SR has been found in cognitive tasks where auditory noise improved the speed of arithmetic computations [19] and recall on visual memory tasks [20]. Thus, adding noise to the input of the information processing system can increase its signal-to-noise output. SR is usually quantified by plotting detection, or cognitive performance, as a function of noise intensity. This relationship follows an inverted U-curve function, where performance peaks at a moderate noise level. That is, moderate noise is beneficial for performance whereas too little, or too much, noise attenuates performance. For extensive reviews on the influence of noise on the nervous system the reader is referred to recent reviews [14,21]. Also detrimental effects of noise on the nervous system and in particular on speech processing are reported in a recent review [22].

### Individual differences in the SR effect

The novel aspect of the proposed framework is that the SR phenomena differs between individuals and these differences are linked to attention ability and neurotransmission in the brain in such way that inattentive persons need more external noise for a proper cognitive functioning. In the model dopamine is the crucial neurotransmitter. This is because it modulates the neural cell's responses to the environment and determines the probability that it will fire following the presentation of a stimulus [23]. Alterations in dopamine function are related to individual differences in attention [24,25], cognition [26] and motivated behavior [27,28]. Dopamine release has both tonic (background levels) and phasic (response to specific environmental events) components regulated by different brain regions [29,30]. Tonic dopamine levels are suggested to modulate the phasic reactivity; a low tonic level increases stimulus dependent phasic release, and the opposite, a high tonic level suppresses phasic release [31]. Low tonic levels cause neural instability associated with cognitive symptoms such as failure to sustain attention [32]. The hypodopaminergic state in ADHD is distinguished by low tonic dopamine levels leading to excessive reactivity to environmental stimulation [33,34]. If the firing probability or gain parameter is low, neurons will fire at random yielding poor cognitive performance. If the gain parameter is high there will be cognitive stability and thus high performance. This responsiveness of neurons is modulated via dopamine that enhances the differentiation between efferent firing and afferent external stimulation. It has been shown recently that neural noise related to dopamine tone is an integral part of inter-neuronal communication and that a sufficient level of noise may be necessary for normal function in the nervous system [21,35], through the process of SR. That is, there exists both external noise - outside of the nervous system - and neural noise (related to dopamine tone) inside the system.

**Figure 1:**
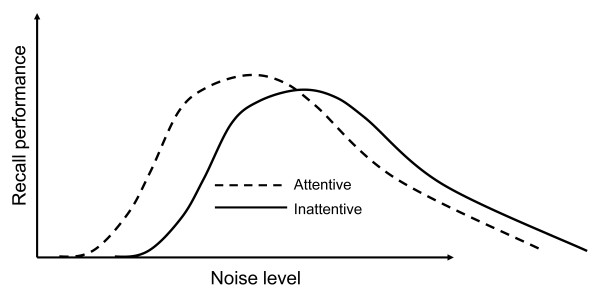
**Individual difference in SR curves**. *Note*. Performance on cognitive tests (y-axis) is optimal for moderate noise levels (x-axis), and attenuated for both too low and too high noise levels. More noise is required for optimal performance in inattentive or low performing children compared to attentive or high performing children.

The moderate brain arousal model (MBA) [13], upon which the current study is based, is a neurocomputational model that relies on classic conditions for stochastic resonance and the modulating properties of dopamine-related gain and neural noise in determining neural responsivity. It suggests that the hypodopaminergic brain need higher input noise to function to its full potential. Thus the model suggests that external white noise could compensate for behavioral dysfunction connected to conditions caused by impaired dopamine transmission. Accordingly, ADHD children or low attentive children more generally have a low gain parameter owing to low levels of baseline dopamine neuron firing. Neurocomputationally the MBA model shows that more external environmental noise is required for optimal performance in cognitive tasks for such low gain "individuals" compared to high gain "individuals". Accordingly, external noise, it is predicted by the model" will compensate for reduced neural background activity in ADHD. That is to say that increased levels of external auditive noise can activate internal noise and restore the activity level. Further, given the inverted U function that operates in relation to noise and performance in SR, the MBA model also predicts that levels of background noise that might be beneficial for ADHD children would be detrimental for those with normal attention. Crucially, the beneficial effects are not specific to ADHD (Figure 1): they are also found in dopamine-related neurodegenerative disorders such as: akinesia [36], Parkinson's disease [37] and in aging [38]. These effects have been modeled in terms of age dependent dopamine loss [39].

In this paper we investigate, for the first time, how noise influences cognitive performance in a normal, non-clinical, group of children that differ from each other in their attentional abilities. Recent evidence suggests that dopamine plays a role in attention also in non-clinical groups. When a visual orienting task was used to study a normal group of children, it was found that those children who were homozygous for alleles influencing dopamine transportation displayed inattention on left-hand sided stimuli, whereas those who were heterozygotes did not [40]. The response to amphetamine has also been found to be influenced by genetic factors; it depends on the functional polymorphisms of the Catechol O-methyltranserase (COMT) gene in a normal population of humans. Mattay et al. [41] found that amphetamine enhanced prefrontal efficiency measured by fMRI for the val/val (high DA metabolism) genotype, whereas met/met (low DA metabolism), had no such effect on low to medium workload, and decreased efficiency on high workload tasks. Furthermore, preservations errors were decreased by amphetamine in the val/val, but not the met/met groups, in a Wisconsin Card Sorting Test (WCST). In another study on healthy controls by Mattay et al. [42] dextroamphetamine was found to increase N-back working memory performance in group with low baseline working-memory capacity, whereas the performance worsened in a group with high baseline performance [42]. Dextroamphetamine also had differential effects on the BOLD fMRI response on these groups. These findings have been corroborated in a study using a spatial working memory task where stimulant medication only improved memory performance in healthy individuals with low baseline working memory capacity [43]. This was also mirrored by increased cerebral blood flow in dorsolateral prefrontal cortex and posterior parietal cortex. Taken together these studies indicate that dopamine function influences performance and brain activity differently also in groups consisting of healthy controls depending on tasks that are linked to attention and working memory (e.g., WCST, N-back, spatial working memory). This body of data is consistent with the view that ADHD and related attentional problems is best conceptualized as a continuum rather than a discrete category and that ADHD symptoms are distributed in populations [44]. The syndrome of ADHD represents a transition of degree rather than of a kind and diagnostic thresholds are therefore somewhat arbitrary resting on general and cultural norms about behavior and development [45]. In addition, a comparison of the extreme points in a normal distribution shows the same heritability patterns as a comparison between ADHD and control [46]. This, in turn, suggests that non-clinical persons with low and high attention may show similar effect from noise as ADHD and control persons do.

In this paper, we study inattentive and normally attentive children's episodic memory in a verb-noun sentence recall task under two conditions varying in terms of the levels of background auditive white noise. Our prediction is that in the low noise condition the inattentive children will perform less well than attentive children while in the high noise condition these differences should diminish as the addition of white noise benefits the inattentive but not the normally attentive children.

## Methods

### Participants

Fifty-one secondary school pupils (25 boys and 26 girls) between 11-12 years (M = 11.7) participated in the study. The group consisted of children from two school classes, year seven (86% of children participated). Participants where divided into two groups after their attention abilities were assessed by teachers using a seven point Likert scale. The assessment scale is the same as that used in the longitudinal research program *Individual Development and Adaptation *[47]. Participants that scored high (6 or 7 - severe problems in the class room) on inattention where assigned to the inattentive group. This group consisted of 10 participants, 6 of them also scored high on hyperactivity as evaluated by the teachers. The comparison group comprised the remaining 41 children that scored 5 or lower at the scale, and were assessed as average- or highly attentive. None of the inattentive group had an ADHD diagnosis and none were treated with medication. Achievements and scholastic skills (3 point scale) and reading ability (7 point scale) used in [47] were also rated by their teachers. Children's school achievement was assessed in terms of whether their school performance was at the level expected for this age group. General cognitive skills and reasoning ability were assessed using the Raven's progressive matrices test [48]. The Raven's test assesses learning and problem solving ability and correlates highly with the g-factor in IQ tests. Forward and backward digit span measuring short-term and working memory respectively were assessed. Table 1 shows the results from these tests for the high and low attentive groups as well as other background characteristics of these groups. The groups were well matched on school performance and general cognitive ability - the inattentive group had less developed reading skills.

**Table 1:** Participant characteristics and cognitive test scores

Cognitive and behavioral measures	Attentive group N = 41 *(20 boys, 21 girls)*		Inattentive group N = 10 *(5 boys, 5 girls)*		Attentive vs. Inattentive
	**Mean SD**	**Range**	**Mean SD**	**Range**	**t-test score**

Inattention (1 = low, 7 = high)	2.8 (1.6)	1 - 7	6.2 (0.4)	1 - 7	t(49) = 6.67***

Hyperactivity (1 = low, 7 = high)	2.5 (1.6)	1 - 7	4.9 (2.0)	1 - 7	t(49) = 4.11***

School performance (1 = below, 2 = average, 3 = above)	2.3 (0.7)	1 - 3	2.1 (0.7)	1 - 3	t(49) = 0.94

Reading skill (1 = low, 7 = high)	5.0 (2.0)	1 - 7	3.5 (1.7)	1 - 7	t(49) = 2.38*

Raven score	41.2 (8.6)	16 - 55	37.3 (10.6)	19 - 53	t(49) = 1.09

Digits forward	23.1 (9.1)	4 - 42	15.0 (6.6)	4 - 23	t(49) = 3.22**

Digits backwards	14.9 (6.9)	2 - 34	13.2 (4.2)	7 - 18	t(49) = 0.97

Note: * p < .05, ** p < .01, *** p < .001

### Design

We used a 2 × 2 design, where noise levels (low versus high) was the within participant manipulation and the between group variable was teacher rated classroom attention level (normal versus inattentive).

### Materials

All participants undertook a verbal episodic recall test. The to-be-remembered (TBR) items consisted of 96 sentences divided into 8 separate lists with 12 verb-noun sentences in each list. Each sentence consisted of a unique verb and a unique noun (e.g., "roll the ball") in Norwegian. The sentences were placed in random order. List-order (1-8) and condition-order (no noise vs. noise) were counterbalanced and noise was present on every second list. All to-be-remembered sentences were recorded on a CD. A new item was read every 9th second. The sentences were read in both the low noise and the high noise condition. The equivalent continuous sound level of the white noise was 78 and the speech signal was 86 dB; thus the signal-to-noise ratio was 8 dB. The signal was sufficiently strong so that all participants could perceive the content of the words in both conditions without error (i.e., the tests were a cognitive memory test and not a perceptual test). The two noise levels were chosen to correspond to levels that have been found in earlier studies to affect cognition in an arithmetic's test for a normal population [19] and working memory performance in patients with Alzheimer's disease [4]. Recordings were made in a sound studio.

### Procedure

The testing was conducted at the child's school, following permission from parents and children. The University College of Sogndal, Norway and the regional ethic board in Stockholm approved the study. The participants were tested individually in a room during the school day. The test lasted for about 45 minutes including the presentation of instructions. Before starting the experiment proper, two practice sentences were presented. The time taken to present each list was approximately 1 minute and 40 seconds. The to-be-remembered sentences were presented concurrent with continuous white noise during the encoding phase in the high noise condition and in silence in the low noise condition. Noise conditions changed after every sentence list in outbalanced order. No noise was presented during retrieval. Directly after presentation of the last item in a list, participants performed a free recall test in which they spoke out loud as many sentences as possible, in any order.

## Results

### Recall performance

**Figure 2:**
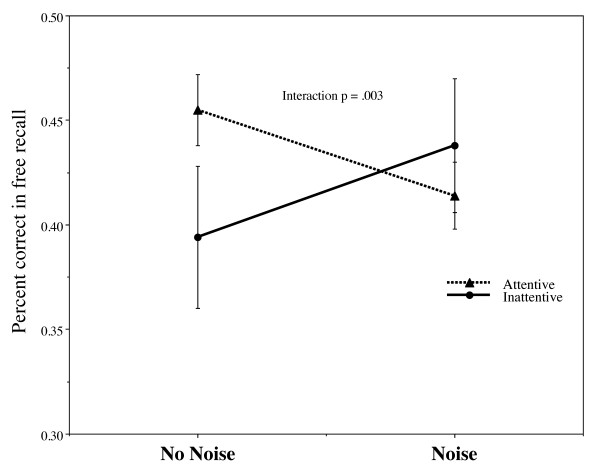
**Recall performance as a function of noise and attention; inattentive vs. attentive children (teachers judgments: attentive N = 41; inattentive: N = 10)**.

A 2 × 2 mixed ANOVA was conducted with one between-subject factor, Group (normal attention vs inattentive) and one within-subjects factor, encoding condition (low noise vs high noise). We choose the standard scoring procedure in the action memory literature [10,49], where strict scoring is used for the nouns (exact matches were required) and lenient scoring is used for verbs (where non-exact matches are scored as correct). This is because nouns are typically recalled somewhat more easily than verbs [50]. There where no main effects of noise (*F*(49,1) = .01, *p *= .94) or group (*F*(49,1) = .30, *p *= .59). Both groups performed at the same level overall across the two conditions. However, the interaction between noise and group was significant (*F*(49,1) = 9.96, *p *= .003, eta^2 ^= .17) (Figure 2). This interaction was further explored with planned simple contrasts for the within subjects factors, using paired sample t-tests. Consistent with the hypothesis the addition of white noise enhanced performance for the inattentive group (M = .39 vs. .44), and impaired performance for the attentive group (M = .46 vs. .41). Inattentive children performed better in the high rather than the low noise condition (*t*(9) = 1.84, *p *= .05 one-tailed). The opposite was the case for the normally attentive group (*t*(40) = -3.46, *p *= .001). Using attention (score 1-7) and noise-effect (noise - no noise) as variables a Spearman rank-order correlation revealed positive correlation between attention and noise (*r *= .378, N = 51, *p *= .006; a medium effect size according to Cohen's d). The higher score on inattention the larger was the positive effect of noise and vice versa, attentive children performed worse in presence of noise.

As reported above the two groups differed with respect to reading skills, as judged by teachers; with the inattentive group having inferior reading skills compared to the attentive group. However, when reading ability was added as a covariate to the main analysis the original interaction effect, although diminished, persisted (F(48,1) = 7.13, *p *= .010). Reading level was independently related to performance (F(6,1) = 1.46, p = .213). A Spearman's rank order correlation revealed negative correlation between reader skills and positive effect of noise (*r *= .335, N = 51, *p *= .016), a positive correlation between attention and reading ability (*r *= .498, N = 51, *p *< .001) and finally, a high positive correlation between teacher ratings of inattention and hyperactivity (*r *= .789, N = 51, *p *< .001). However, there was no correlation between hyperactivity and noise-effect (*r *= .141, N = 51, *p *= .323).

## Discussion

We have proposed a framework for understanding individual difference in the facilitative effects of auditive white noise on performance. As predicted the results show different effects of noise in attentive and inattentive children selected from the normal population. There was significant improvement in performance for the children rated as inattentive by their teachers, and a significant decline in performance for those rated as attentive as noise levels were increased. Furthermore these effects seem independent of other factors measured in the study - attentional ability seems to be a key marker of this effect. Even if inattention and hyperactivity are strongly correlated, no correlation between hyperactivity and a positive noise effect was found, this suggests that in this study inattention is the key factor to explain noise improvement. These results are similar to those previously reported with ADHD patients [10]. Here we discuss theoretical and practical implications of these findings.

From a theoretical point of view the findings are consistent with the suggestion that the neural noise level associated with dopamine tone in inattentive children is sub-optimal, see also [13] and that noise may enhance performance through the phenomenon of stochastic resonance (SR). According to the model developed here, noise in the environment yields an input to the perceptual system, which can either compensate for low noise in the neural system leading to an output consisting of improved cognitive performance, or, depending on pre-existing levels of neural noise, can add too much to an already well functioning system. The specific neuro-biological and neuro-chemical mechanisms responsible for these effects need further research. For instance, auditive white noise may have its effects either at a perceptual or neuro-psychological level or may operate a neuro-chemical level directly altering levels of dopamine release [13,51]. Animal models of dopamine function or pharmacological probes to manipulate tonic and phasic dopamine are called for to investigate these effects.

Stimulant medication (e.g. methylphenidate) also improves cognitive performance in children with ADHD [52,53]. This medication increases dopamine levels by blocking the dopamine transporter [54]. Low performing healthy controls also benefit from increased dopamine transmission, which is manifested in improved cognitive performance and increased prefrontal cortical activity [43]. Our data show that auditory white noise may exert potentially similar effects on cognition as medication through the phenomenon of stochastic resonance (SR). White noise is characterized by randomness and so introduces variability in the nervous system [14]. A poorly tuned neural system benefit from additional white noise. In fact, the stochastic resonance theory predicts that noise that is applied to the signal as an input to a neural cell, improves the signaling efficiency of the output of that cell, where the non-linearity in the firing threshold of the neural cells is the key to improvement of the signal to noise ratio [14,55].

Despite the fact that it appears that noise and methylphenidate both improve cognitive performance, the underlying mechanism that is the basis of these phenomena are likely to be different. According to the model theoretically speaking the difference between these phenomena is clear. Methylphenidate changes the strength (but not the variability) of the input, which is typically modeled by the gain parameter in abstract neural networks [23]. In contrast, noise changes the variability of the input (but per definition does not influence the strength) of the input. However, despite these clear differences in the underlying level, the behavioral outcome may be similar, and the two mechanisms interact in a complex way, making it difficult to distinguish the phenomena at the behavioral level [56]. Furthermore, direct evidence of difference between these levels is emerging. Pålsson and Söderlund et al. (Noise benefit in pre-pulse inhibition of the acoustic startle reflex, submitted) studied the effect of methylphenidate, dopamine and noise on the startle response in a rat model of ADHD. They found that both control and ADHD strains (SHR) benefited from noise; however, this effect was also found in dopamine lesioned rats, suggesting that dopamine is not a necessary requirement for the stochastic resonance phenomenon to occur.

Another theoretical interpretation of the data is that noise in a general way increase arousal that makes the subject more alert, and less drowsy. The optimal stimulation model states that hyperactivity is as a homeostatic response to underarousal in order to achieve an optimal arousal level [11]. However, this model does not make any explicit predictions about the selective effects of external stimulation whereas in the current study inattentive persons benefited from extra stimulation and attentive children did not. In the cognitive energetic model state factors like arousal, activation and effort are taken into account to explain shortcomings in ADHD patients [57]. According to this model state factors can be moderated by event rate (inter-stimulus-intervals, ISI) and workload in cognitive tasks both under- and over arousal can be produced. Recent research has shown that stimulant medication (methylphenidate) and shortened event rate can produce the same effect in a Go-Nogo task [58]. From our point of view the term arousal is poorly defined in the literature, and could be interpreted in terms of wakefulness or in term of neural arousal. To fully investigate the arousal-noise hypothesis an experiment would have to be designed where physiological arousal is explicitly manipulated and measured. We argue that that proposed framework, including the dopaminergic influence on stochastic resonance, provides a more elaborated view both at the neural and at the behavioral level. To account for the current data an arousal view would have to argue for a selective lower arousal for the inattentive children. Finally, our experience is that the subjects in our experiment are fully aroused, the testing conditions at hand are very stimulating, and subjects are very motivated to perform well.

By highlighting the role of individual differences in the facilitative effects of auditive noise the current study refines our understanding of SR. SR exhibits an inverted U-curve function, where performance peaks at a moderate noise level. However, this is an oversimplification, as there is no absolute sense in which a moderate noise level is optimal. An "optimal" noise level for one individual could be either too high, or too low amount of noise for another individual. These complex interactions between noise and performance may account for some of the contradictory findings in the previous literature. For example, earlier research on noise in normal populations has shown both enhancing and diminishing effects of auditory white noise on cognition in non-clinical groups (90 dB) on simpler, short-term memory tasks like anagrams, whereas speech noise was detrimental [59]. These noise effects also interacted with other variables such as gender and time of the day [60], which makes these results equivocal. No effect of white noise was found in digit span recall in two experiments, whereas speech noise had a detrimental effect [4,5]. However, noise improvement was found in a simple addition task in selected groups, elderly and young participants [61] and among elderly and Alzheimer patients [4]. In Broadbent's early research negative effects of noise have been found using high (excessive) noise levels around 100 dB [62-64]. In later experiments by Broadbent and colleagues, (memory recall of unrelated words) lower noise levels (80-85 dB) were used; results showed no effects of noise on memory when exposed during the encoding phase but deteriorating results if exposed during the recall phase [65,66]. More recently, episodic memory has been found to be particularly vulnerable to speech noise, whereas traffic noise showed no effect [2]. Results from the present study would indicate that the effects of external noise would have look quite different in many of these studies if participants had been divided into attentive and inattentive or young and elderly, that is high and low gain participants (tentatively high/low dopamine groups). Selective effects of noise can easily get hidden in group-means if some participants improve and others are impaired. Our data may encourage noise researchers to reanalyze their experiments dividing participants by individual differences in attention and performance. Preliminary data from our lab, on an ADHD rat model provide further support for the benefits of adding white noise. The Spontaneous hypertensive rat (SHR) showed improved sensorimotor gating by showing more a pronounced pre-pulse inhibition of the startle reflex when exposed to white noise as compared to control strains, even though control rats also increased their inhibition in noise conditions (Pålsson, Söderlund et al., Noise benefit in pre-pulse inhibition of the acoustic startle reflex, submitted).

Reading disability is a common co-morbidity in ADHD. Consistent with these findings, our data show lower reading skill for the inattentive group. Reading disability is also linked with reduced short-term verbal memory that requires phonetic coding of material, but not necessarily with executive functions or long-term memory [67,68]. This is also consistent with our data that show a lower performance in the digits forward task that measures short-term memory, but no deficits for digits backward task that is related to working memory capacity. The MBA model accounts for these findings because the digits backward task is a more demanding task leading to higher brain arousal and thus good performance for inattentive children, whereas the less demanding digit forwards task does not sufficiently arouse the brain for the low attention children [13]. Additionally, the positive correlation between reading ability and noise enhancement suggests that white noise may enhance awareness. This is consistent with the idea that dyslexia is caused by phonological deficits [69], however, a further investigation of phonologic awareness is outside the scoop of the present study.

## Limitations

The current study has a number of limitations. First, ratings of academic attainment and reading were based on single item non-validated scales. Second, only two noise levels were investigated. It would be interesting to include different levels so that the entire stochastic resonance curve can be mapped out. One prediction from our model is that in attentive group, a subset of participants will benefit from noise when levels are individually adjusted. Furthermore, it would be of interest to investigate if the SR effect is dependent on task difficulty. Third, only one test of cognitive ability was tested. Future research may study whether the effects found here generalizes to other tasks such as executive and inhibitory functions. This is of particular importance while investigating the possibility of noise, as an intervention in ADHD. A final limitation of the present study is that the experiment was not designed to study differential effects of noise on study and recall. The experiment manipulates noise at encoding, but not at retrieval, so the effects of noise seen can only be attributed to the conditions at encoding. To what extent noise presented at recall influences performance cannot be determined by the experiment. However, the MBA predicts that the conditions where noise is beneficial during encoding should also be beneficial for performance during retrieval. At same time it may be difficult to directly compare encoding and retrieval conditions, because the task demand for encoding task may differ from the demands at retrieval.

## Conclusions

In summary, the present study suggests that cognitive performance can be moderated by external stimulation in a non-clinical group of teacher-rated inattentive participants. If replicated this finding could have practical applications offering a non-invasive help to improve school results in children with attentional problems. In particular awareness should be raised regarding the possibility that the environment has be individually adjusted to the need of the children, where inattentive children in a normal population show noise benefit when performing cognitive tasks. In our data these effects eliminated the differences between high performing, attentive and low performing inattentive children. The possibility that attention can be improved by the addition of carefully controlled levels of white noise into ones environment is potentially of major practical significance. Currently ADHD children are treated successful with medication, where environmental stimulation could be seen as a complementary method to deal with inattentive problems. This could be of particular importance for the significant population of parents that are uneasy about the use of medication.

## Competing interests

The authors G. Söderlund, S. Sikström and, J-M. Loftesnes declare that they have no competing interest.

Financial disclosures and conflicts of interest statement E. Sonuga-Barke:

Recent speaker board: Shire, UCB Pharma

Current & recent consultancy: UCB Pharma, Shire

Current & recent research support: Janssen Cilag, Shire, Qbtech, Flynn Pharma

Advisory Board: Shire, Flynn Pharma, UCB Pharma, Astra Zeneca

Conference support: Shire

## Authors' contributions

GS planned and designed the experiment, performed the statistical analysis and drafted the manuscript. SS have been involved in drafting and revision of the manuscript. LML was responsible for the data collection. ESB have been involved in revising the manuscript critically for important intellectual content. All authors read and approved the final manuscript.
